# Osteoporosis and Celiac Disease: Updates and Hidden Pitfalls

**DOI:** 10.3390/nu15051089

**Published:** 2023-02-22

**Authors:** Lisa Lungaro, Francesca Manza, Anna Costanzini, Marianna Barbalinardo, Denis Gentili, Fabio Caputo, Matteo Guarino, Giorgio Zoli, Umberto Volta, Roberto De Giorgio, Giacomo Caio

**Affiliations:** 1 Department of Translational Medicine, University of Ferrara, 44121 Ferrara, Italy; 2 National Research Council, Institute for the Study of Nanostructured Materials (CNR-ISMN), 40129 Bologna, Italy; 3 Department of Medical and Surgical Sciences, University of Bologna, 40138 Bologna, Italy; 4 Mucosal Immunology and Biology Research Center, Massachusetts General Hospital—Harvard Medical School, Boston, MA 02114, USA

**Keywords:** celiac disease, osteoporosis, osteopenia, bone mineral density, management of osteoporosis, bone–gut axis, sex-related differences

## Abstract

Celiac disease (CD) is an autoimmune disorder caused by gluten ingestion in genetically predisposed individuals. In addition to the typical gastrointestinal symptoms (e.g., diarrhea, bloating, and chronic abdominal pain), CD may also present with a broad spectrum of manifestations, including low bone mineral density (BMD) and osteoporosis. The etiopathology of bone lesions in CD is multifactorial and other conditions, rather than mineral and vitamin D malabsorption, may affect skeletal health, especially those related to the endocrine system. Here, we describe CD-induced osteoporosis in an attempt to enlighten new and less-known aspects, such as the influence of the intestinal microbiome and sex-related differences on bone health. This review describes the role of CD in the development of skeletal alterations to provide physicians with an updated overview on this debated topic and to improve the management of osteoporosis in CD.

## 1. Celiac Disease: An Introduction

CD is an autoimmune condition triggered by gluten ingestion in susceptible subjects that primarily affects the proximal small intestine. Genetic background is an essential prerogative for the development of the disease (HLA-DQ2/DQ8 positivity and non-HLA genes); however, the contribution of other factors (e.g., viral infections and gut dysbiosis) might also play a role [1].

Many studies [2,3,4] indicate that gluten is a digestion-resistant protein formed by several immunogenic peptides that can trigger the host’s immunity responses, increasing gut permeability and innate and adaptive immune system activation, stimulating IL-8 and TNF-α production.

Until a few decades ago, CD was considered an uncommon disease affecting mainly children and European-native individuals [5]. Nowadays, CD is considered a global burden, since its prevalence reaches almost 1% of the worldwide population, making it one of the most common autoimmune disorders [6]. Consequently, the growing attention paid to the disease means that it is more frequently diagnosed in both the pediatric population and adults, especially throughout industrialized countries, as described in a recent meta-analysis by King et al. [7]. In addition, the increased number of diagnosis depends on the increasing incidence rates [8]. Based on the “hygiene hypothesis” [9], better hygienic conditions in Western countries led to a reduction in the incidence of infections and therefore promotion of allergic and autoimmune diseases. This might explain the rise in CD cases along with the high consumption of gluten in our diets [10]. More recently, Fasano et al. suggested that an increase in intestinal permeability (also referred to as ‘leaky gut’) and dysbiosis play a key pathogenic role in CD [11]. Indeed, the impaired expression of zonulin causes an abnormal antigen trafficking from the lumen to the lamina propria, triggering innate and immunoregulatory responses that lead to a pro-inflammatory micromilieu.

CD is diagnosed two to three times more frequently in women than men. The disease can occur at any age, from early childhood to the elderly, with two peaks of onset, one shortly after weaning with gluten in the first two years of life and the other in the second or third decades [1]. According to the 2012 Oslo classification, the latest consensus that categorizes CD nosology, the disease may be classified as classical, non-classical, subclinical, potential and refractory. The classical form of CD shows signs and symptoms of malabsorption, i.e., diarrhea, steatorrhea, weight loss, or growth failure, and it is more frequent in the pediatric population. The non-classical CD is characterized by constipation, alternating bowel habit, osteopenia/osteoporosis, and recurrent miscarriages. The term “subclinical” identifies a form of CD below the threshold of clinical detection without signs or symptoms sufficient to trigger CD serology to be tested in daily practice. Refractory CD affects patients that present persistent symptoms and / or signs of malabsorption despite strict gluten withdrawal for at least 12–18 months. Finally, the term “potential CD” describes a normal small intestinal mucosa with positive CD serology [12]. These patients are at an increased risk of developing overt CD; however, if patients are asymptomatic, GFD should not be mandatorily prescribed [13].

The European Society for the Study of Celiac Disease (ESsCD) guidelines suggest that IgA-transglutaminase type 2 (IgA-TG2) antibody is the best single test for CD detection at any age, along with total IgA levels, which should be always assessed to rule out IgA-deficiency, which occurs in up to 8% of CD patients [14]. It is of paramount importance that all serologic tests are performed while patients are on a gluten-containing diet [15]. In adults, the gold standard for CD diagnosis is the combination of serological test (i.e., anti-TG2, anti-endomysium, and deamidated gliadin peptide antibodies) and mucosal changes detected by duodenal biopsy according to Marsh-Oberhüber or Corazza and Villanacci classifications [16]. A simple rule by Catassi and Fasano suggests that the diagnosis of CD is confirmed if at least four of the following five criteria are satisfied: (1) typical symptoms of celiac disease; (2) positivity of serum celiac disease IgA class autoantibodies at high titer; (3) HLA-DQ2 or DQ8 genotypes; (4) celiac enteropathy at the small intestinal biopsy; and (5) response to the GFD (three out of four if HLA is not performed) [17]. To date, the current standard of care is based on these criteria [1].

Currently, the only effective treatment for CD is a lifelong strict gluten-free diet (GFD), leading to the resolution of intestinal and extraintestinal symptoms, negativization of autoantibodies, and regrowth of the intestinal villi [1].

The etiopathology of osteoporosis in CD is multifactorial, and in the past years, most of the mechanisms involved in the process have been unveiled. Nowadays, medicine has taken the direction of a more personalized approach, and emerging topics such as gut microbiome and sex-related differences are becoming pivotal. Thus, we aim to provide clinicians with an up-to-date overview of old and new pathological and clinical aspects to improve the management of osteoporosis in CD.

## 2. Methods

In this narrative review, we conducted a PubMed, EMBASE, MEDLINE, and ScienceDirect search from the inception of January 2013 to the end of December 2022 using the following search terms: “celiac disease”, “osteoporosis”, ”osteoporosis AND celiac disease”, ”osteoporosis guidelines”, ”gut microbiota AND bone”, “gut microbiota AND celiac disease”, “probiotics AND celiac disease”, ”secondary osteoporosis”, “male osteoporosis”, “celiac disease AND growth”, “endocrine axis AND celiac disease”, “osteoporosis treatment” and related results.

The following criteria restricted the search strategy: (1) reported bone-associated conditions in CD; (2) papers published in the last ten years (January 2013–end of December 2022); (3) papers written in English; and (4) full text available. The article search was carried out independently by two authors (LL and FM) who screened the titles and abstracts of the selected records. The records identified at the first step were then thoroughly evaluated, considering the manuscript and appendices. Non-pertinent papers or those not matching the inclusion criteria were excluded. Duplicates, papers with no original data, and incomplete or unclear outcomes were also excluded.

## 3. Pathologic Bone Alterations in Celiac Disease

Extraintestinal manifestations (EIM) are common in CD and include: abnormal liver enzymes, arthralgia/arthritis, dermatitis herpetiformis, alopecia, fatigue, headache, anemia, stomatitis, myalgias, psychiatric disorders, rashes, seizures, neuropathy, short stature, delayed puberty, and infertility [18]. A well-established relationship between low bone mineral density (BMD) and CD has been determined [19]. Pathologic bone alterations include osteopenia and osteoporosis due to malabsorption (leading to calcium and vitamin D deficiencies) and chronic inflammation with the secretion of pro-inflammatory cytokines. Hence, CD is considered a secondary osteoporosis risk factor [20]. Osteoporosis is characterized by a significant loss of bone mass affecting skeletal tissue density and architectural rearrangement both contributing to an increased risk of fractures as well as an increased morbidity and mortality rate [21]. Bone loss starts at the age of 35, but it reaches its peak in late middle life (50 > years) and affects mainly women [22]. In the absence of an underlying disease, osteoporosis is defined as “primary”, whereas it is labeled “secondary” if the result of a condition or medication [23]. 

A recent meta-analysis reports that 30–60% of newly diagnosed patients with CD show low BMD and 18–35% osteoporosis, implying that bone alterations are extremely frequent in CD [24,25,26]. However, there is still a lack of univocal conclusion: Stenson et al. [27] found a 3.4% incidence of CD in osteoporotic patients compared to 0.2% among the general population, while others found increased IgA EmA values in sera of osteoporotic patients with normal intestinal mucosa [28,29].

In some cases, it has been reported that patients could show osteoporosis with elevated serum alkaline phosphatase levels (ALP) and hyperparathyroidism as the sole presentation of CD [30]. Walker et al. suggested that forearm bone density measurement, usually not screened when assessing BMD, is of aid to identify osteoporosis; it may be performed in addition to hip and lumbar spine BMD evaluation, which alone tends to underestimate the prevalence of osteoporosis [31].

### 3.1. Mechanisms Underlying Osteoporosis in Celiac Disease

**Figure 1:**
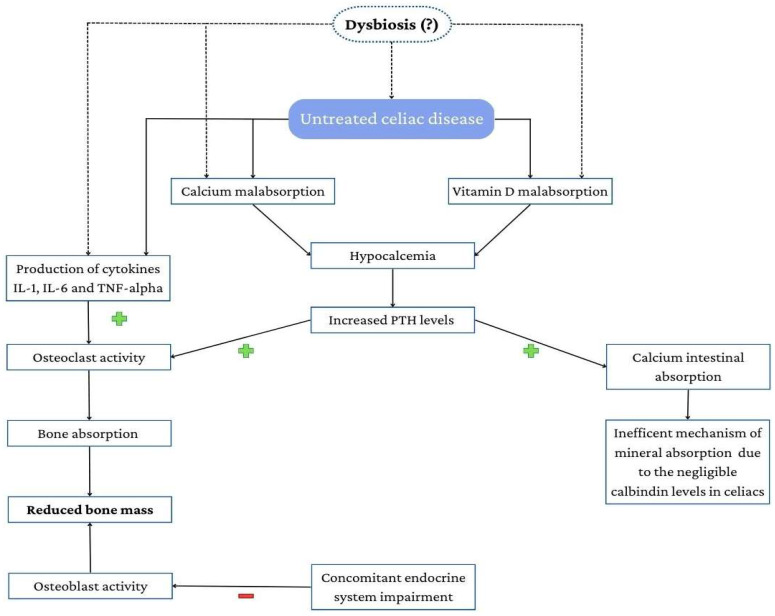
Possible mechanisms involved in bone demineralization in CD. The presence of dysbiosis alone, even in the absence of villous atrophy, can induce calcium and vitamin D malabsorption.

Bone is a mineralized tissue that undergoes continuous remodeling, which is determined by the synergic action of osteoblasts and osteoclasts and regulated by a complex interaction of different mechanisms. The peak of bone mass is reached at the age of 20–25, whereas in the third decade of life, the process of bone reabsorption begins to exceed bone formation, leading to progressive bone loss. Hormones and nutrition play an important role in this process, and they may be impaired in CD. Figure 1 summarizes the possible mechanisms involved in bone demineralization in CD [32].

#### 3.1.1. Calcium Homeostasis

Calcium balance is regulated through a complex and coordinated action of hormones (parathyroid hormone PTH, 1,25-dihydroxyvitamin D and calcitonin) and organs: bones, as a calcium reservoir, intestine, which regulates the exogen absorption, and kidneys. As blood Ca^2+^ concentration decreases, a rapid increase in PTH release promotes bone turnover and cortical bone loss. Thus, Ca^2+^ malabsorption in CD plays a pivotal role in the induction of a series of events that lead to bone demineralization [25]. Hyperparathyroidism is frequent and should be sought in newly diagnosed patients as it is responsible for accelerating bone turnover [33,34]. In CD, the histological damage of villi in proximal intestinal mucosa impairs Ca^2+^ absorption [35].

Low calcium intake is a risk factor for osteoporosis, and calcium intake in the young age is an essential determinant of the bone mass peak [36,37]. Calcium metabolism defects are common in untreated children with CD, and they return to normal after GFD [38]: indeed, adherence to strict GFD for at least 1–2 years is sufficient to normalize Ca^2+^ and vitamin D levels. However, there are some cases (e.g., post-menopausal women) where diet is insufficient to replenish minerals and vitamins, and low BMD becomes chronic. In these subsets of patients, long-term vitamin D and calcium supplementation are recommended [39].

Industrial gluten-free products could have low levels of minerals, including Ca^2+^, and patients might not assume the recommended daily intake (RDA), suggesting the introduction of fortified gluten-free foods or calcium supplementation [40].

#### 3.1.2. The Role of Vitamin D

Vitamin D contributes significantly to bone mineralization and the maintenance of calcium and phosphate homeostasis. It regulates intestinal calcium intake through vitamin D receptor (VDR), normally expressed in CD patients, without any significant difference compared to that of controls [41]. Vitamin D (as well as calcium) is often malabsorbed in untreated CD due to villous atrophy and steatorrhea [33]. Furthermore, vitamin D is activated by the PTH-mediated enzyme 1-alpha-hydroxylase activation in the kidney. In CD, this signal promotes the intestinal absorption of calcium through an increased vitamin D-dependent active transport. However, even if enterocytes have an average number of vitamin D receptors, they present negligible amounts of vitamin D-dependent calcium-binding protein (calbindin) due to their immaturity, thereby making this mechanism ineffective. Moreover, high levels of 1,25-vitamin D may, paradoxically, exert a negative effect on the skeleton, causing bone resorption [24]. Furthermore, vitamin D acts directly on the immune system, thus playing an important role in autoimmune diseases. Clinical studies have demonstrated that vitamin D deficiency is related to morbidity in infectious diseases and the onset or progression of autoimmune diseases, including CD [42,43,44].

Vitamin D deficiency is more prevalent in adult and pediatric patients with CD than controls [45]. Insufficient dietary intake and impaired intestinal absorption are considered to be crucial factors in the development of vitamin D deficiency. Mautalen et al. [46] found that calcium and vitamin D supplementation did not provide additional benefit to that obtained by diet alone. Accordingly, Barera et al. [47] reported only a marginal effect of low-dose vitamin D supplementation, resulting in a rise of solely vitamin D levels but no significant changes in BMD. As for calcium, a strict adherence to GFD normalizes vitamin D levels in most CD patients. Moreover, some studies suggest that most patients with intestinal inflammatory diseases (e.g., CD, Crohn’s disease, enteropathies), regardless of their condition, have normal intestinal vitamin D absorption but are vitamin D deficient. In contrast, some patients have normal serum vitamin D levels, even though their intestinal vitamin D absorption is severely impaired [48,49]. This discrepancy suggests that other factors, such as insufficient light exposure, inflammation, and hyperparathyroidism, significantly affect vitamin D serum levels [50].

### 3.2. Sex Differences

A systematic review [51] analyzed osteoporosis and osteopenia incidence among both premenopausal females and males with CD, confirming the association between CD and the increasing risk of fractures. It also stated the need to consider confounding factors such as sex, reproductive status, age, smoke, steroid exposure, and endocrine disorders when describing CD patients. Meyer et al. [52] evaluated 105 women and 23 men with CD who BMD measured, finding out that men, compared to age-matched controls, are affected by a more severe form of low BMD than both pre- and post-menopausal women. In addition, this study confirms that most papers either investigate women only, without considering differences among sexes, or, if considered, the results were similar in both populations. Despite the low number of male patients, the results of this study were consistent with those reported by Bayer et al. [53]. They analyzed BMD at the lumbar spine of 42 celiac children (23 girls and 19 boys) who were not taking any medication. Mean BMD was significantly lower in boys than girls in comparison to control groups. Moreover, girls with menarche had a higher BMD than prepubertal ones, but this result did not reach statistical significance. Both studies agreed that endogenous estrogens in women might be at least a partial protecting factor for bone health in CD. While in women with CD, there is a clear association between the reduction in estrogens during menopause and the development of osteoporosis [54], in men, there are no certain connections between low BMD and the effect of other factors, such as growth hormone (GH) and hypogonadism, besides nutrients, and calcium and vitamin D deficiencies due to villous atrophy [55].

Overall, celiac women are at a higher risk of osteoporosis for indirect effects (early menopause, amenorrhea) and direct ones (malabsorption). Early menopause should draw attention to lifestyle changes (e.g., smoking cessation, doing physical exercise, assuming dietary supplements) and, if needed, the administration of hormone replacement therapy is suggested [55].

### 3.3. Endocrine Axis

Children with short stature should be investigated for CD [56]. Indeed, the short height has been traditionally considered a natural consequence of malabsorption since “catch-up growth” (i.e., a much faster height gain than average) is a common finding after the beginning of a GFD [57]. Although most of the studies reported that CD children following a GFD reach a final stature in line with the general population [58,59], interestingly, Weiss et al. [60] and Sonti et al. [61] found that delayed diagnosis of CD may lead to a shorter adult height in men but not in women. Furthermore, it is known that CD is associated with immune-mediated endocrine axis impairment (e.g., autoimmune thyroiditis, diabetes mellitus type 1 (DMT1)) [62], with some new data revealing a connection between CD and GH deficiency (GHD). Giovenale et al. [63] studied 7066 pediatric patients with short stature: 44 of them were diagnosed with CD, 650 were diagnosed with GHD, and an association of GHD and CD was found in 16 children. Despite suggestive results [64], we still need more confirmation to assess a precise connection between CD and GHD. Delvecchio et al. [65] found a remarkable prevalence of positive anti-pituitary antibodies (APA) in newly diagnosed CD patients. High APA titers are associated with height impairment, which is likely mediated by a reduction in insulin-like growth factor (IGF-1), suggesting that an autoimmune pituitary process could also induce a linear-growth impairment. Since GH is pivotal for bone formation via IGF-1, acting directly on osteoblast receptors [23], it is clear that a lack of this hormone, particularly in CD, might act as a precipitating factor for the development of osteoporosis. Several studies [56,66] have reported normalization of growth in children after GH replacement therapy. However, there is still a lack of data concerning bone health in patients with both CD and GHD, and more studies are needed to confirm this association [67]. The coexistence of autoimmune thyroid diseases (AITD), DMT1 and CD has been assessed [68,69], and they are both known to be causes of secondary osteoporosis. It is reasonable to suppose that patients affected by CD and DMT1/AITD have an increased risk of osteoporosis than CD patients only, even though more research on the topic is needed to define this dependency properly.

Untreated CD patients present higher serum prolactin (PRL) levels [70]. Despite the immune-modulator action, PRL impacts bone metabolism, contributing to low BMD and osteoporosis [71]. Moreover, high PRL negatively affects gonadal function in both sexes [72,73], acting as an additional risk factor for osteoporosis.

### 3.4. The Emerging Role of Gut Microbiota

Emerging studies describe the role of gut microbiota in bone health. The human gut contains 100 trillion microbial cells, which are now known to play a significant role in modulating the adaptive immune system [74,75]. Moreover, the gut microbiota regulates mineral absorption, including calcium, phosphorous and magnesium uptake, and bone turnover, through gut-derived factors, such as incretins, serotonin, and IGF-1. These are only a few of the many different mechanisms that have been proposed to explain how gut microbiota impacts on skeletal homeostasis [76]. Furthermore, it has been recently demonstrated that even in the absence of villous atrophy, osteopenia and osteoporosis can occur, which is possibly due to a mild mucosa inflammation associated with intestinal dysbiosis. In this line, a paper by Carroccio et al. [77] highlighted a higher frequency of osteopenia and osteoporosis in patients with non-celiac gluten/wheat sensitivity (NCG/WS), which is not related with the degree of the inflammation of the duodenal mucosa. Indeed, people with NCG/WS do not have villous atrophy and overt malabsorption (duodenal biopsy usually is normal—Marsh 0—or in about 40% of cases displaying a mild inflammation—Marsh 1). The alteration of the intestinal barrier function induced by dysbiosis could be a possible pathogenetic mechanism of this gluten-related disorder inducing calcium malabsorption and bone alterations [78].

Different bacterial strains might likely act via separate and overlapping pathways on bone health [79]. Several studies on murine models support this strong relationship: McCabe et al. [80] administered *Lactobacillus reuteri* ATCC 6475 to healthy male mice and demonstrated a significant increase in trabecular bone density, thickness and mineral content compared to untreated controls. Other studies described a beneficial effect on rat bone health markers after the treatment with *Bifidobacterium longum* [81], *Lactobacillus rhamnosus* [82], and *Lactobacillus paracasei* [83].

In humans, there are just a few pieces of evidence supporting the role of gut bacteria: a mix of *Lactobacilli,* including *L. reuteri*, seems to prevent bone loss at the distal tibia, at the lumbar spine or at hip, with an improvement comparable to the group treated with calcium ± vitamin D [84,85]. A double-blind, placebo-controlled randomized controlled trial observed that the assumption of *L. casei Shirota* accelerates functional recovery in patients with distal radius fracture rather than in those assuming placebo [86].

*Lactobacilli* and *Bifidobacterium* spp. are also thought to play a role in the breakdown of gluten and in the enhancement of mucosal barrier: CD patients have shown a reduction in these beneficial species in different studies [87,88]. A recent meta-analysis of randomized clinical trials [89] examined more than 2000 literature records on probiotics and CD and included only seven of them in a quantitative synthesis. Different probiotic strains might have various effects on manifestation related to gluten exposure (e.g., intestinal inflammation, mucosal barrier integrity, symptoms related to accidental gluten ingestion); they are safe and may be beneficial in a subset of patients [90]. All the studies we analyzed highlighted the need for further high-quality research on probiotics in CD and on the “gut–bone axis”, which are still relatively novel concepts that need additional rigorous inquiry, especially regarding the mechanism underneath. To date, there are no studies establishing a clear correlation between CD, low BMD, and probiotics, albeit there might be a common thread and therapeutic implications.

## 4. Management of Bone Disease

A balanced GFD is of pivotal importance to restore mucosal integrity and allowing proper micronutrient absorption, including calcium and vitamin D. Nutritional counseling should be provided by a dietician or nutritionist with expertise in GFD to guarantee adequate nutrients and micronutrient intake. Casella et al. found that T scores improved significantly after GFD (from 1.64 ± 1.07 to 1.40 ± 1.07 at the lumbar-sacral spine and from 1.25 ± 0.62 to 1.04 ± 0.74 at the femoral neck) [91], which is a result confirmed by other studies [92,93,94], making GFD alone effective for the improvement of BMD.

Calcium, PTH, vitamin D, and ALP should be assessed at the time of the diagnosis. A study by Tanenbaum et al. confirmed the importance of laboratory testing in the evaluation of secondary osteoporosis, including serum complete blood count, calcium, phosphate, liver function tests, creatinine, albumin/globulin, PTH, 25-OH D, 24 h urine calcium/creatinine ratio, and TSH. They found that 56 out of 173 osteoporotic women in their cohort (32%) had undetected but identifiable and treatable etiologies of secondary bone loss [95].

However, in case of low serum levels, inadequate calcium and/or vitamin D intake, or after a year of GFD without improving BMD, supplementation is recommended [96].

The presence of other concomitant autoimmune diseases should be evaluated at the time of diagnosis of CD; thyroid assets (TSH, thyroid autoantibodies) should be checked every 36 months, and the metabolic profile, including glucose levels, should be checked every year [97]. Furthermore, in children, an inadequate catch-up growth after >12 months on a strict GFD is indicative of a compromised GH status or, in the presence of delayed puberty, of a gonadal impairment. Thus, a prompt evaluation and diagnosis are mandatory to start adequate therapy immediately. Comorbidities represent contributing causes that lead to a higher risk of developing osteoporosis. Assessing whether a strict GFD is followed is important to avoid symptoms misconception and missing diagnoses of other underlying diseases. In addition, education on the importance of lifestyle changes, such as regular exercise, smoking cessation, and reduction in excessive alcohol intake, should be provided [32].

**Figure 2:**
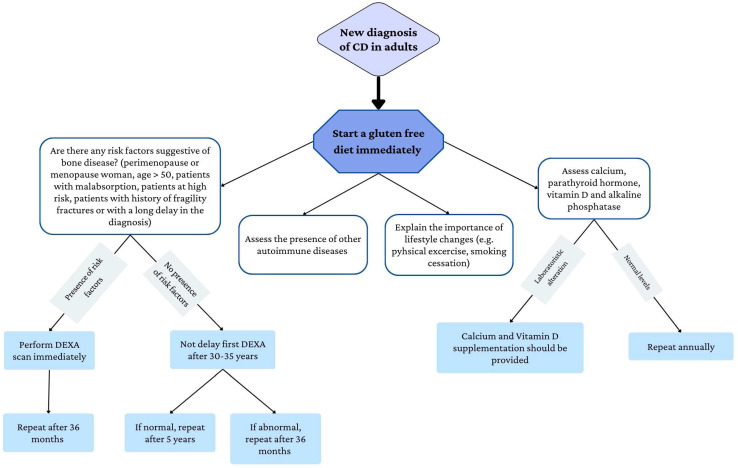
Flow chart of the management of bone health in CD patients.

Both the British Society of Gastroenterology Guidelines for the diagnosis and management of CD [98] and ESsCD recommend a dual-energy X-ray absorptiometry (DEXA) screening in celiac patients, especially in those at high risk of osteoporosis [15]. DEXA should be performed at the time of the diagnosis in high-risk patients, in patients with malabsorption, and those with a long delay in CD diagnosis [99]. Figure 2 below summarizes the principles of the management of bone disease in CD.

## 5. Pharmacological Treatment

Identifying CD patients at high risk of developing osteoporotic fractures is fundamental to treat them with an adequate standard of care and eventually starting a pharmacological treatment. An anti-osteoporotic therapy should aim at reducing the risk of fracture, and it is based on an accurate evaluation of DEXA T and Z score integrated with other important clinical data such as age, steroid therapy, and smoking habit. Those inputs are combined in validated scales such as “Fracture Risk Assessment Tool” (FRAX^®^) or “Derived Fracture Risk Assessment” (DeFRA) to assess the estimated risk of hip or other major fractures in the next 10 years [100]. However, it should be considered that these tools are not validated in the young population. Osteoporosis in patients with CD might be an indication for pharmacological treatment. Nevertheless, osteopenia itself is not enough to begin antiresorptive treatment unless in patients at an increased risk of osteoporosis. Patients should be referred to an endocrinologist or rheumatologist to receive proper counseling.

The currently approved pharmacologic therapeutics for the prevention and/or treatment of osteoporosis in adults include bisphosphonates, estrogens (estrogen and/or hormone therapy), estrogen agonist/antagonist (raloxifene, bazedoxifene/conjugated estrogens), parathyroid hormone (PTH [1,2,3,4,5,6,7,8,9,10,11,12,13,14,15,16,17,18,19,20,21,22,23,24,25,26,27,28,29,30,31,32,33,34], teriparatide), analog of with parathyroid hormone-related peptide (PTHrP [1,2,3,4,5,6,7,8,9,10,11,12,13,14,15,16,17,18,19,20,21,22,23,24,25,26,27,28,29,30,31,32,33,34], abaloparatide), RANKL inhibitor (denosumab), fully human monoclonal antibody to sclerostin (romosozumab), and calcitonin [101] (Table 1).

In children with CD, vitamin D supplementation should be prescribed if: (1) vitamin D levels are lower than 20 ng/mL; (2) vitamin D levels are between 20 and 30 ng/mL showing a Z-score  ≤  −2 (or other data indicative of bone fragility). As well as vitamin D, calcium should be supplemented in children with low BMD or osteoporosis, especially if dietary intake is inadequate. Plasmatic levels of 25-hydroxyvitamin D_3_ should be higher than 30 ng/dL. The treatment with bisphosphonates, which are used off-label in childhood osteoporosis, should be administered in patients with Z-score ≤ −2 and two or more pathological fractures or vertebral fractures regardless of the Z-score, with the previous obtainment of informed consent. Usually, prophylactic bisphosphonate therapy (i.e., treating a low bone density Z-score in the absence of fracture) is not recommended [103]. 

In men with age < 50 years, pharmacological treatment is recommended in the presence of a recent fragility fracture or Z-score < −2; in men older than 50 years, treatment is recommended in the presence of a recent fragility fracture, T-score < −2.5 (the number of standard deviation units in relation to the young reference healthy population), or T-score < −2 in the presence of diabetes if the patient is assuming glucocorticoid or an androgen deprivation therapy or if indicated by FRAX [105].

Albeit progress, univocal conclusions on the definition of osteoporosis and its treatment in premenopausal women and young adults are still missing. It has been suggested that a T-score below −2.5 at the spine or hip should be considered as a diagnostic of osteoporosis if the patient suffers from a chronic disorder known to affect bone metabolism, such as CD [107]. Most authorities recommend conservative therapy since the risk of fractures is relatively rare, and its reduction is not granted with a pharmacological intervention. Primarily, vitamins and nutritional deficiencies should be targeted. The treatment of the underlying cause of osteoporosis is strongly recommended, but when it is not possible, it may require a prescription (estrogens, bisphosphonates, denosumab, or anabolic agents, such as teriparatide) [108].

However, clinical evidence on anti-osteoporotic drugs, including bisphosphonates, comes mainly from studies on post-menopausal women who are noticeably at a higher risk of osteoporosis. One pilot study assessed BMD changes after treatment with the addition of zoledronic acid compared to the standard treatment group (GFD + calcium carbonate + cholecalciferol). The rise in BMD was higher in the drug arm compared to the control group but did not achieve statistical significance [109]. Overall, data concerning the efficacy of pharmaceuticals in CD patients and other subpopulations still need to be made available, and there is an eagerness for more studies on the topic.

## 6. Conclusions

Osteopenia and osteoporosis are conditions frequently associated with CD. Strict adherence to GFD seems to be the only effective treatment to improve BMD in adults and normalize BMD in children [110]. However, patients’ nutritional, metabolic, and endocrinologic status should also be considered. There is an eagerness for more studies to assess further the link between osteoporosis in CD and sex-related differences, which might help develop a tailored approach to the patient. There is little evidence regarding osteopenia and pharmacological osteoporosis treatment, specifically in CD. Probiotic supplementation might become a novel strategy in preventing bone alterations, albeit the role of gut microbiota is still uncertain and not well-established yet. Physicians should be aware of bone conditions linked to CD that might contribute to the worsening of BMD and should treat them immediately. A more careful approach in at-risk populations would ensure earlier diagnosis and a more targeted therapy.

## Figures and Tables

**Table 1:** Currently approved pharmacological agents used for adult and pediatric osteoporosis.

Pharmacological Therapeutics for Osteoporosis			
Medication		Mechanism of Action	Target Groups
**Biphosphonates:**	*Alendronate*	Bisphosphonates have an affinity with hydroxyapatite, and they inhibit farnesyl pyrophosphate synthase in osteoclast. They induce osteoclasts’ apoptosis, inhibiting bone resorption and increasing BMD [102].	Women, men, off-label use in children [103]
	*Risendronate*		
	*Ibandronate*		
	*Pamidronate*		
	*Zolendronate*		
**Monoclonal antibodies:**	*Denosumab*	Monoclonal antibody to the RANKL, a major regulator of bone resorption.	Women and men
	*Romosozumab*	Monoclonal antibody to sclerosin, a blocking protein to the WNT bone signaling pathway. A strong anabolic drug which improves bone formation and suppresses resorption [104].	Post-menopausal women only
**Anabolic agent:**	*Teriparatide*	It is a portion [1,2,3,4,5,6,7,8,9,10,11,12,13,14,15,16,17,18,19,20,21,22,23,24,25,26,27,28,29,30,31,32,33,34] of human PTH. Intermittent exposure to PTH will activate osteoblasts more than osteoclasts. Thus, once-daily injections of teriparatide have been shown to reduce significantly the risk of vertebral fractures and also non-vertebral fractures [102].	Women and men [105]
	*Abaloparatide*	It is a parathyroid hormone 1 receptor (PTHrP) analogue; it selectively activates the PTH1 receptor, a G protein-coupled receptor (GPCR) expressed in the osteoblasts and osteocytes [106].	Post-menopausal women only
**Estrogen-related drugs:**	*Raloxifene*	Selective estrogen receptor modulators (SERMs), hence mixed agonist and antagonist of the estrogen receptor in different tissues. In bone, they have an estrogenic (protective) activity.	Post-menopausal women only [102]
	*Bazedoxifene/* *conjugated estrogens*		
	*Hormone replacement therapy*	It mimics the protective effects of estrogens and progesterone. It prevents bone loss in post-menopausal women by inhibiting osteoclast-driven bone resorption and reducing the rate of bone remodeling [102].	

## Data Availability

Not applicable.
